# Acute Physiological Responses to Resistance Exercise With Continuous Versus Intermittent Blood Flow Restriction: A Randomized Controlled Trial

**DOI:** 10.3389/fphys.2020.00132

**Published:** 2020-03-17

**Authors:** Eduardo D. S. Freitas, Ryan M. Miller, Aaron D. Heishman, João B. Ferreira-Júnior, Joamira P. Araújo, Michael G. Bemben

**Affiliations:** ^1^Neuromuscular Laboratory, Department of Health and Exercise Science, University of Oklahoma, Norman, OK, United States; ^2^Kinanthropometry and Human Performance Laboratory, Federal Institute of Sudeste of Minas Gerais, Rio Pomba, Brazil; ^3^Kinanthropometry and Human Performance Laboratory, Department of Physical Education, Federal University of Paraíba, João Pessoa, Brazil

**Keywords:** muscle activity, electromyography, lactate, muscle swelling, kaatsu, occlusion training, strength training

## Abstract

The primary goal of this investigation was to examine the physiological responses of blood flow restriction (BFR) resistance exercise (RE) performed with continuous or intermittent BFR and to compare these results to those from conventional high- and low-load RE without BFR. Fourteen men randomly completed the following experimental trials: (1) low-load RE with continuous BFR (cBFR), (2) low-load RE with intermittent BFR (iBFR), (3) low-load RE without BFR (LI), and (4) conventional high-load RE without BFR (HI). For the cBFR, iBFR, and LI exercise trials, participants performed four sets of 30–15–15–15 repetitions of the bilateral leg press (LP) and knee extension (KE) exercises, at an intensity of 20% of their one-repetition maximum (1-RM), at a 1.5-s contraction speed, and with a 1-min rest period between sets. The only difference between the cBFR and iBFR protocols was that the pressure of the cuffs was released during the rest intervals between sets for the iBFR trial. For the HI trial, participants completed four sets of 10 repetitions of the same exercises, at 70% of 1-RM, with a 1-min rest period between sets, and at the same contraction speed. Muscle activity was assessed during each set using superficial electromyography, as well as changes in blood lactate concentration [La^–^] from baseline at 5 min post exercise and in muscle swelling and plasma volume (%ΔPV) at 5 and 15 min post exercise. There were no significant differences in muscle activity (*p* < 0.05) across the cBFR, iBFR, and LI protocols at any time point, whereas they were all significantly lower than HI. There were also no significant (*p* < 0.05) differences across the three low-load RE conditions for [La^–^],%ΔPV, or muscle swelling. HI elicited significantly (*p* < 0.05) greater responses than cBFR, iBFR, and LI for all the physiological markers measured. In conclusion, RE combined with cBFR or iBFR induce the same acute physiological responses. However, the largest physiological responses are observed with HI, probably because of the significantly greater exercise volumes. Therefore, releasing the pressure of the restrictive cuffs during the rest periods between sets will not hinder the acute physiological responses from BFR RE.

## Introduction

Low-load resistance training combined with blood flow restriction (BFR) has challenged traditional beliefs that loads superior to 65% of one-repetition maximum (1-RM) are required to elicit significant increases in muscle size and strength (American College of Sports Medicine [ACSM], 2011). In fact, previous studies have demonstrated that BFR resistance exercise is capable of eliciting muscle hypertrophy gains and muscle function improvements across a variety of populations (Takarada et al., 2000b; Yasuda et al., 2014; Buford et al., 2015; Jørgensen et al., 2015; Tennent et al., 2017). The inclusion of training loads as low as 20–30% of 1-RM has made BFR resistance exercise alluring as a potential training alternative to conventional high-load resistance training, which may benefit those unable to lift heavy loads.

Although it has been documented that conventional low-load resistance exercise performed to volitional failure may also induce muscle hypertrophy gains and improve muscle function (Mitchell et al., 2012; Ogasawara et al., 2013), large exercise volumes need to be achieved, making this training approach impractical. On the other hand, in addition to low loads, BFR resistance training also utilizes low exercise volumes and still has been shown capable of increasing muscle cross-sectional area in a similar fashion to high-load resistance training (Vechin et al., 2015). The underlying mechanisms responsible for the chronic adaptations following BFR resistance training remain unclear. However, it has been speculated that it may be due to the activation of the type II muscle fibers (Fatela et al., 2016), the accumulation of metabolites within the intramuscular environment (Suga et al., 2010), anabolic hormone secretion (Takarada et al., 2000a), exercise-induced muscle swelling (Freitas et al., 2017), and the regulation of biomolecular pathways (Fujita et al., 2007; Nakajima et al., 2016).

Blood flow restriction resistance exercise induces local hypoxia by the placement of pressurized cuffs at the proximal portion of the muscle, which reduces arterial inflow and impedes venous return, thus resulting in venous pooling (Iida et al., 2011). The restrictive cuffs are commonly inflated at the beginning of the exercise bout and, then, only deflated following exercise completion, leading to BFR resistance exercise commonly resulting in considerable local discomfort (Hughes et al., 2018). Therefore, it has been hypothesized that releasing the pressure of the cuffs during the rest periods between sets, also known as intermittent BFR, may attenuate discomfort and increase exercise tolerability (Manini et al., 2012). However, it is unknown how this approach would affect the acute physiological responses commonly observed with BFR resistance exercise that are thought to be involved in the training hypertrophic response.

Previous research has investigated the physiological responses to intermittent BFR resistance exercise; however, flaws in the research design of these studies have limited the interpretation of their results (Suga et al., 2012; Yasuda et al., 2013; Fitschen et al., 2014). For instance, Yasuda et al. (2013) observed no difference in muscle activity during either continuous or intermittent BFR resistance exercise, but the authors failed to individualize the restrictive pressure applied to the participants in the study. Current guidelines recommend making the BFR pressure relative to each individual (Scott et al., 2014; Patterson et al., 2019). Utilizing a fixed restrictive pressure would lead to different levels of restriction across participants and potentially lead to distinct acute physiological responses, hence increasing data variability and compromising the research design. Additionally, previous studies investigating the physiological responses to BFR resistance exercise have utilized unilateral exercises and small muscle groups or deviated from the current standard of four sets of 30–15–15–15 repetitions (Suga et al., 2012), commonly used throughout the literature. To illustrate, Suga et al. (2012) had participants perform three sets of 30 repetitions of unilateral plantar flexion. Utilizing such a small muscle group limits the exercise-induced metabolic responses, thus limiting the ability of the investigator to detect potential differences across exercise protocols, in addition to deviating from real-life gym settings where individuals usually exercise using larger muscle groups in multi-joint exercises.

Therefore, the purpose of the current investigation was to examine the acute physiological responses of young males to resistance exercise performed with continuous and intermittent BFR, utilizing larger muscle groups, two lower-limb exercises, and individualized restrictive pressures. Lower-body exercises were chosen because they recruit larger muscle mass, which may potentially elicit greater physiological responses. Moreover, the individuals’ physiological responses to both BFR resistance exercise protocols were compared to those from traditional resistance exercise performed with low and high loads. It was hypothesized that continuous BFR exercise would elicit greater physiological responses compared to intermittent BFR exercise, considering that deflation of the restrictive cuffs during the rest intervals between sets may impede the accumulation of metabolites in the intramuscular environment. Our secondary hypothesis was that the physiological responses observed with both continuous and intermittent BFR would be lower compared to those from traditional high-load resistance exercise, considering previous evidence that higher mechanical stress tends to induce greater metabolic responses (Schoenfeld, 2013), but higher than those from traditional low-load resistance exercise without BFR, due to the lack of the BFR stimuli.

## Materials and Methods

### Participants

Fourteen young males aged between 18 and 30 years volunteered for the current study. Participants were normotensive, free from any osteomuscular or cardiovascular diseases, not involved in any resistance exercise program over the past 6 months, not taking any medications, and had an ankle–brachial index between 0.9 and 1.20. Participants refrained from caffeine for at least 6 h and strenuous exercise or alcohol for at least 24 h prior to each experimental session. All participants were provided verbal explanations about all tests and procedures, and informed written consent was obtained prior to any participation. This study was performed in accordance with the Declaration of Helsinki and approved by the University of Oklahoma Institutional Review Board. This study is part of a major project that also investigated the perceptual responses to both forms of BFR resistance exercise (Freitas et al., 2019).

### Study Design

This study consisted of a randomized within-within subject crossover design that compared the acute physiological responses to continuous and intermittent BFR resistance exercise, while also comparing these results to those from conventional high- and low-load resistance exercises. Participants attended the laboratory on six different occasions. During the first visit, participants consented, filled out standardized questionnaires, and completed a 1-RM test. During the next visit, participants’ body composition was assessed using a total-body dual-energy X-ray absorptiometry (DXA) scan, the 1-RM test was performed a second time, and participants were familiarized with the exercise procedures. During the last four visits, participants were required to randomly complete each one of the following exercise trials: (1) low-load resistance exercise with continuous BFR (cBFR), (2) low-load resistance exercise with intermittent BFR (iBFR), (3) low-load resistance exercise without BFR (LI), and (4) high-load resistance exercise without BFR (HI). Muscle activity was assessed during each set of exercise, as well as changes in blood lactate concentration, muscle swelling, and plasma volumes changes.

### Body Composition

DXA (Luna Prodigy DXA, Healthcare, Madison, MI, United States) scans were used to assess participants’ body composition. Whole-body scans were performed to estimate bone-free lean body mass, fat mass, and bone mineral content. Before each scan, participants were asked to remove their shoes and any metal accessories (e.g. earrings, necklace, and piercings) and to wear minimal clothing. During the scans, participants lied down in the supine position, with arms and legs straight, and head positioned 2 to 3 cm below the horizontal line at the top of the measuring table. Hips and shoulders were evenly spaced in the center of the table with arms close to the body without touching it. Straps were positioned at the knees and ankles and were used to prevent movement and to keep the legs straight during the scan. Quality assurance tests were performed at each testing day for calibration and to ensure that the device was working properly. All scans were analyzed by the same trained technician using specialized software (enCORE 16, Healthcare, WI, United States).

### Maximum Dynamic Strength Test

Participants’ maximum dynamic strength was assessed using bilateral 1-RM tests for the leg press (LP) and knee extension (KE) exercises following the National Strength and Conditioning Association’s guidelines (Baechle and Earle, 2016). Before initiating the test, each participant was introduced to the proper technique and completed the first warm-up, which consisted of performing 8 to 10 repetitions with a moderate to light load. Following a load increment, the participant was asked to complete four to five repetitions. Then, the load was increased once again, and the participant completed two to three repetitions. After the warm-up, the load was progressively increased until the participant was unable to complete a repetition using proper form and technique. The 1-RM for all participants was determined within three to five attempts. Participants were allowed 3 min to rest between the LP and KE tests. The load lifted during each experimental trial was determined based on each participant’s highest 1-RM value for each exercise. Test–retest reliability information regarding the 1-RM test has been reported elsewhere (Freitas et al., 2019). The same investigator performed all the 1-RM tests for each participant in order to avoid any inter-tester variability.

### Assessment of Total BFR Pressure

The restrictive pressure applied during the two BFR experimental trials was individually determined for each participant and based on the total arterial occlusion pressure for the lower body. After arriving at the laboratory, participants lied down in the supine position and rested for 10 min; then, a portable automatic monitor (BP710, OMRON, IL) was used to assess the brachial arterial pressure. After that, a 13.5-cm-wide nylon cuff (SC12, D.E. Hokanson, Bellevue, WA, United States) connected to a rapid inflator system (E20 Rapid Cuff Inflator, D. E. Hokanson, Bellevue, WA, United States) was placed close to the inguinal fold region of the thigh and inflated to 50 mmHg for 30 s, while a handheld bidirectional Doppler probe (MD6 Doppler, D. E. Hokanson, Bellevue, WA, United States) coated with transmission gel was positioned over the posterior tibial artery to detect the auscultatory pulse. Then, the cuff was inflated to the participant’s systolic blood pressure, measured in the arm, for about 10 s. Then, the cuff was deflated, and cycles of inflation and deflation were performed with progressive intervals of 10 mmHg, until the auscultatory pulse was completely interrupted. Once the auscultatory pulse could no longer be detected by the Doppler, the pressure was slowly released until it could be redetected. The restrictive pressure required to fully interrupt the auscultatory pulse to the lower body was considered the total occlusion pressure. The same procedures were repeated in the contralateral limb and the average occlusion pressure of both limbs was used to calculate the 50% restrictive pressure to be used during the two BFR experimental protocols. The average total occlusion pressure was 139.75 ± 14.41 mmHg.

### Resistance Exercise Protocols

Participants were required to randomly complete each one of the following exercise conditions: (1) low-load resistance exercise with continuous BFR (cBFR), consisting of four sets of 30–15–15–15 repetitions of the bilateral horizontal LP and KE exercises, always in this order, at an intensity of 20% of 1-RM and with 50% of BFR, meaning that the cuffs remained inflated during the entire exercise period and that they were only deflated during the 3-min interval between exercises; (2) low-load resistance exercise with intermittent BFR (iBFR), which was identical to the cBFR protocol, except that the pressure of the cuffs was released during the 1-min rest interval between sets; (3) low-load resistance exercise without BFR (LI), which was equivalent to the cBFR and iBFR protocols, but with no restriction of blood flow; and (4) high-load resistance exercise (HI), which included four sets of 10 repetitions, performed at an intensity of 70% of 1-RM, with 1-min rest interval between sets and 3-min between exercises. For both cBFR and iBFR trials, the same 13.5-cm-wide pneumatic cuffs used to determine the total occlusion pressure were positioned at the inguinal crease region of each thigh and utilized to reduce blood flow during exercise. The contraction speed was standardized at 1.5-s for each portion of the contraction and controlled using a digital metronome, set at 40 beats per minute. There was a washout period of 3 to 7 days between each trial, and participants were not tested if they reported that any level of soreness from a previous testing visit was still present. Each testing session was performed at the same time of the day (±1 h) to minimize variation due to the circadian rhythm.

### Muscle Activity

Muscle activity was assessed during each experimental trial in the muscle vastus medialis and vastus lateralis of the dominant leg using superficial electromyography (EMG). Bipolar electrodes (EL503, Biopac System, Inc., Goleta, CA, United States) were placed at the belly of each muscle with a 20-mm distance between electrodes, in accordance with SENIAM’s recommendations. For the vastus medialis, the electrodes were placed at 80% of the distance between the anterior spina iliac superior and the joint space in front of the anterior border of the medial ligament. For the vastus lateralis, electrodes were placed at two thirds of the distance between the anterior spina iliac superior to the lateral side of the patella. A semipermanent ink was used to mark the sites for initial electrode placement in an attempt to guarantee that electrodes were placed at approximately the same locations during each experimental trial. The electrodes were connected to an amplifier and digitized system (MP 100, Biopac System, Inc., Goleta, CA, United States), while a ground electrode was placed at the calcaneal protuberance of left ankle. The signal was captured at a frequency of 1,000 Hz, amplified 1,000 times, and stored in a portable computer for analysis using the AcqKnowledge software (AcqKnowledge 3.8.1, Biopac System, Inc., Goleta, CA, United States). Before analysis, the signal was filtered using low- and high-pass filters of 500 and 10 Hz, respectively. Normalization was performed using the signal obtained during a maximum voluntary dynamic contraction (MVDC) equivalent to the participants’ 1-RM load and performed immediately before each experimental trial. The EMG signal obtained during each MVDC was utilized to determine EMG reliability. The intraclass correlation coefficients (ICC) for muscle activity for the LP and KE MVDCs were 0.95 and 0.90, respectively. To determine muscle activity during each experimental condition, the concentric portion of each individual muscle contraction of each set was analyzed using root mean squares. The concentric portions were isolated from the eccentric portions using the event markers function available on the AcqKnowledge software. Strokes on the F9 key on the computer keyboard performed by the investigator during exercise would add markers alongside the EMG signal that would identify the beginning and the end of each concentric and eccentric contraction. After analysis, each set was divided into three portions: initial, middle, and final portions. Each portion of the set consisted of an average of 10 contractions for the sets with 30 repetitions and the average of five contractions for the sets with 15 repetitions. For the sets with 10 repetitions, the initial, middle, and final portions were given as the average of the first three, middle four, and last three contractions, respectively.

### Blood Lactate

Whole-blood lactate [La^–^] concentration was measured immediately before, immediately post, and 5 min post exercise for all four trials utilizing a portable lactate analyzer (Lactate Plus, Nova Biomedical Corporation, Waltham, MA, United States) and lactate test strips (Lactate Plus, Nova Biomedical Corporation, Waltham, MA, United States). Blood samples of about 5 μl were collected through finger pricks performed in the index or middle fingers. Before the blood was collected, the finger was swiped with alcohol, and the first drop was discarded. The lactate analyzer was calibrated every day before data collection using low and high lactate standards (Lactate Plus, Nova Biomedical Corporation, Waltham, MA, United States), following the manufacture’s recommendations. [La^–^] levels were posteriorly corrected for plasma volume shifts using the following equation:

$${\left[{\text{La}}^{-}\right]}_{\text{c}}={\left[{\text{La}}^{-}\right]}_{\text{un}}\times \left(\frac{100+\%\mathrm{PV}\Delta }{100}\right)$$

here [La^–^]_*c*_ stands for corrected lactate concentration, [La^–^]_*un*_ stands for uncorrected lactate concentration, and %PVΔ stands for percent changes in plasma volume.

### Muscle Swelling

**FIGURE 1:**
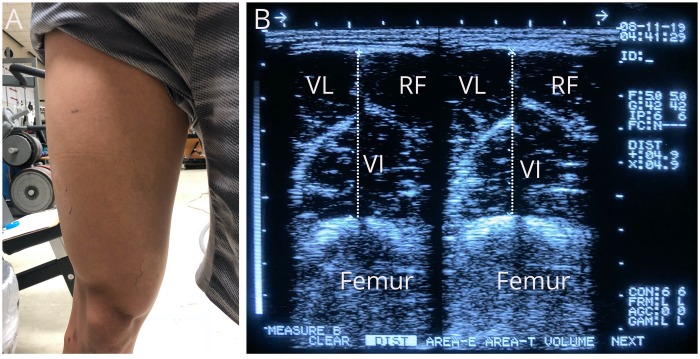
**(A)** Fifty percent thigh site used for muscle thickness assessment. **(B)** Image of a muscle thickness measurement performed in duplicate. VL: Vastus lateralis, RT: Rectus femoris, VI: Vastus intermedius.

Muscle swelling was estimated using muscle thickness and thigh circumference measurements performed at the 50% femur site of the dominant leg immediately before, immediately post, 5 min post, and 15 min post exercise. Muscle thickness was assessed utilizing an ultrasound machine (FF Sonic UF-4500, Fukuda Denshi, Tokyo, Japan) and a 5-MHz scanning head, coated with transmission gel and placed at the 50% femur site (i.e. the halfway point between the lateral condyle of the femur and the great trochanter) as displayed in Figure 1A. Muscle thickness consisted of the perpendicular distance between the adipose tissue–muscle interface and the muscle–bone interface (Figure 1B). Thigh circumference was also measured at the same site following each muscle thickness assessment. Both muscle thickness and thigh circumference assessments were performed in duplicate by the same trained technician and averaged at each time point. The 50% thigh site was marked with a semipermanent ink to ensure consistency of the measurements across the different testing visits. The ICC values within and between measures were 0.99 and 0.98, respectively, for muscle thickness and 0.99 and 0.99, respectively, for thigh circumference. The minimal differences needed to be considered a real change within and between measures were 0.21 and 0.57 cm, respectively, for muscle thickness and 0.63 and 1.29 cm, respectively, for thigh circumference.

### Hematocrit Levels and Plasma Volume Changes

Hematocrit levels (Hct) and percent changes in plasma volume (%ΔPV) were assessed immediately before, immediately post, 5 min post, and 15 min post exercise. Following sterilization, the participants’ index or middle finger was pricked, and a small blood sample was collected into a heparinized plastic microhematocrit tube, which was centrifuged to separate Hct from plasma. Hct-to-plasma percentage was determined in each sample using a micro-capillary reader (Damon/IEC Division, Needham, MA).%ΔPV were calculated using the equation below (Van Beaumont, 1972), previously used by our research group (Freitas et al., 2017):

$$\%\,\Delta \,\mathrm{PV}=\left(\frac{100}{100-{\mathrm{Hct}}_{\mathrm{Pre}}}\right)\times 100\times \left(\left(\frac{{\mathrm{Hct}}_{\mathrm{Pre}}}{\left({\mathrm{Hct}}_{\mathrm{Pre}}-{\mathrm{Hct}}_{\mathrm{Post}}\right)}\right)/H\,c\,t_{\mathrm{Post}}\right)$$

Hct and %ΔPV were determined in duplicate by the same trained technician. The ICCs within measures for Hct and %ΔPV were 0.94 and 0.81, respectively.

### Statistical Analyses

Data normality was determined using the Shapiro–Wilk test and graphical information. One-way repeated-measures analyses of variance (ANOVAs) were used to test differences in total exercise volume across conditions. Two-way (condition × time) repeated-measures ANOVAs were used to test all main effects and interactions. Whenever a significant interaction effect was observed, the model was decomposed, and a separate one-way repeated-measures ANOVA was carried out. Muscle activity between the vastus medialis and vastus lateralis muscles was compared during each set of each exercise condition during LP and KE using a three-way (muscle [2] × set [4] × exercise condition [4]) mixed factorial model. Muscle activity of the two muscles was then averaged and analyzed using a three-way (condition [4] × set [4] × portion of the set [3]) mixed factorial model. In the case of significant interactions, simple effects were analyzed using separate simple one-way ANOVAs and one-way repeated-measures ANOVAs. If the assumption of sphericity was not met, the Greenhouse–Geisser correction was used. Partial eta squared (η*_*p*_*^2^) values were calculated for all main effects and interaction as an estimate of effect size. The Bonferroni procedure was utilized to minimize the familywise error rate whenever pairwise comparisons were performed. Main effects were only interpreted if significant interactions were absent. Sample size was estimated *a priori* using unpublished data from our laboratory collected on 29 participants, utilizing G^∗^Power 3.1 (Franz Faul, University of Kiel, Germany) and using the following data: variance explained by the special effect = 85.066, variance within group = 99.413, η*_*p*_*^2^ = 0.461, effect size = 0.925, α = 0.05, β = 0.8, and correlation among variables = 0.5. ICC estimates were calculated based on an absolute-agreement, two-way mixed-effects model (Koo and Li, 2016). ICC values were used to calculate the standard error of measurement as $S\,E\,M=S\,D\times \sqrt{1-I\,C\,C}$ and the minimal difference (MD) needed to be considered a real change as $M\,D=S\,E\,M\times 1.96\times \sqrt{2}$ (Weir, 2005). SPSS Statistics v.24 (International Business Machines Corp., Armonk, NY, United States) was used for data analysis. All data are presented as mean ± standard deviation, and the level of significance was set at α ≤ 0.05.

## Results

### Participants’ Characteristics and Total Exercise Volume

Table 1 displays the descriptive statistics of the characteristics of all participants included in the study. Total exercise volume was calculated as load × repetitions × sets completed for each exercise condition for the LP and KE exercises. During LP, the HI protocol (6,675.84 ± 1,250.01 kg) resulted in a significantly (*p* < 0.001) greater volume than cBFR (3,129.30 ± 585.94 kg), iBFR (3,129.30 ± 585.94 kg), and LI (3129,0.30 ± 585.94 kg). Similar results were observed during KE, with HI (2,052.32 ± 582.66 kg) resulting in significantly (*p* ≤ 0.01) greater exercise volume than that observed with cBFR (1,428.29 ± 261.82 kg), iBFR (1,429.16 ± 271.07 kg), and LI (1,440.94 ± 260.63 kg). There were no significant (*p* > 0.05) differences in exercise volume across the cBFR, iBFR, and LI trials, during LP or KE.

**TABLE 1:** Descriptive statistics of the characteristics of the study participants.

**Variable**	**Mean ± SD**
Age (years)	21.79 ± 2.97
Weight (kg)	71.60 ± 10.95
Height (m)	1.78 ± 0.06
Body mass index (kg/m^2^)	22.64 ± 3.36
Fat mass (kg)	17.00 ± 6.92
Bone-free lean mass (kg)	51.82 ± 6.11
Bone mineral content (kg)	2.79 ± 0.32
Body fat percentage (%)	23.16 ± 13.48
Leg press 1 RM (kg)	203.15 ± 38.65
Knee extension 1 RM (kg)	95.97 ± 17.36
Total arterial occlusion pressure (mmHg)	139.75 ± 14.41

### Muscle Activity

Since no significant muscle-by-set (LP: *F* = 0.068, *p* < 0.977, KE: *F* = 0.112, *p* < 0.953) or muscle-by-condition (LP: *F* = 0.251, *p* < 0.860, KE: *F* = 0.121, *p* < 0.948) interactions or muscle main effects (LP: *F* = 0.180, *p* < 0.675, KE: *F* = 0.096, *p* < 0.760) were observed during LP and KE in our three-way mixed model comparing the activity of the vastus medialis and vastus lateralis muscles, the muscle activity of these two muscles was averaged and used for data analysis.

**FIGURE 2:**
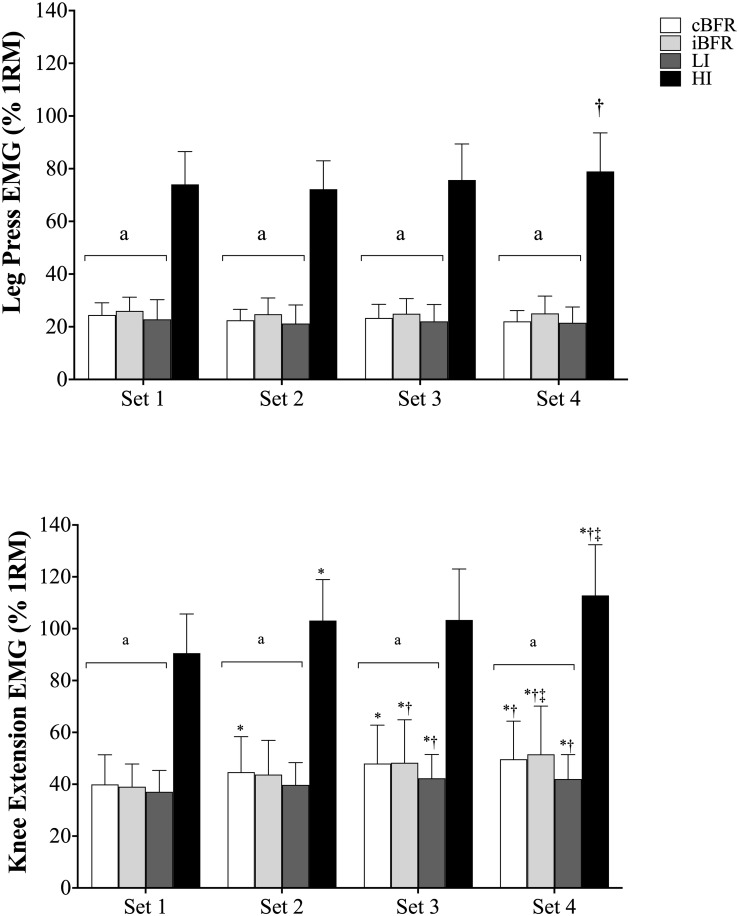
Muscle activity averaged across all repetitions for each set of **(1A)** leg press and **(1B)** knee extension. cBFR, Low-load resistance exercise with continuous BFR; iBFR, low-load resistance exercise with intermittent BFR; LI, low-load resistance exercise without BFR; HI, high-load resistance exercise without BFR. ^a^Significantly lower than HI. ^∗^Significantly greater than set 1. ^†^Significantly greater than set 2. ^‡^Significantly greater than set 3.

Table 2 outlines the changes in muscle activity across the initial, middle, and final portions of each set for all exercise trials, and Figure 2 displays the changes in muscle activity for each set (averaged across all three portions of the set) for all exercise conditions.

**TABLE 2:** Changes in muscle activity [% of one-repetition maximum (1-RM)] within sets of both leg press (LP) and knee extension (KE) exercises across all experimental conditions.

	**Initial portion**	**Middle portion**	**Final portion**
**Leg press**			
cBFR	21.53 ± 3.52	22.80 ± 3.51^b^*	24.91 ± 5.53^b^*
iBFR	23.01 ± 5.43	25.91 ± 6.49^b^*	26.72 ± 5.68^b^*
LI	20.43 ± 6.28	22.50 ± 6.73^b^*	22.82 ± 6.79^b^*
HI	70.48 ± 10.78	74.27 ± 12.19*	81.00 ± 15.19*†
**Knee extension**			
cBFR	39.50 ± 12.86	44.74 ± 13.37^b^*	52.45 ± 15.09^b^*†
iBFR	39.22 ± 13.18	44.99 ± 14.48^b^*	52.80 ± 16.06^b^*†
LI	34.54 ± 7.37	40.40 ± 9.32^b^*	46.00 ± 10.34^b^*†
HI	91.59 ± 13.48	103.99 ± 16.96^†^	111.84 ± 19.27*†

cBFR: low-load resistance exercise condition with continuous blood flow restriction (BFR), iBFR: low-load resistance exercise condition with intermittent BFR, LI: low-load resistance exercise condition without BFR, HI: high-load resistance exercise condition without blood flow restriction. ^*b*^Significantly lower than HI. * Significantly greater than the initial portion. ^†^Significantly greater than the middle portion.

For LP (Table 2 and Figure 2A), there were significant condition-by-set (*F* = 4.36, *p* = 0.01, η*_*p*_*^2^ = 0.27) and condition-by-portion of the set (*F* = 10.11, *p* < 0.001, η*_*p*_*^2^ = 0.46) interactions. Further analyses revealed that there were no significant (*p* > 0.05) differences in muscle activity during LP across the cBFR, iBFR, and LI exercise conditions during all four sets or during the individual portions of each set; however, all the low-load exercise conditions displayed significantly (*p* < 0.05) lower muscle activity compared to the HI condition. For the analyses across sets within each experimental trial (Figure 2A), similar levels of muscle activity (*p* > 0.05) were detected from set 1 to set 4 for the cBFR, iBFR, and LI trials. Similar results were observed for HI, except that set 4 was significantly (*p* < 0.05) greater than set 2. For the comparisons across the initial, middle, and final portions of each set (Table 2), muscle activity measured during the middle and final portions was significantly (*p* < 0.05) greater than that measured in the initial portion of the set with no significant (*p* > 0.05) differences being detected between the middle and final portions for the cBFR, iBFR, and LI exercise conditions. The same results were observed for the HI trial, except that muscle activity during the final portion of the set was significantly (*p* < 0.05) greater than that observed during the middle portion.

During KE (Table 2 and Figure 2B), there were significant condition-by-set (*F* = 2.98, *p* = 0.003, η*_*p*_*^2^ = 0.19), condition-by-portion of the set (*F* = 5.28, *p* = 0.02, η*_*p*_*^2^ = 0.29), and condition-by-set-by-portion of the set (*F* = 1.80, *p* = 0.03, η*_*p*_*^2^ = 0.12) interactions. Follow-up analyses revealed that no significant (*p* > 0.05) differences were observed across the cBFR, iBFR, and LI exercise conditions from set 1 to set 4 or during the initial, middle, and final portions of the set, although HI elicited significantly (*p* < 0.05) greater muscle activity than all trials during all sets and all portions of the sets.

### Whole-Blood Lactate

**FIGURE 3:**
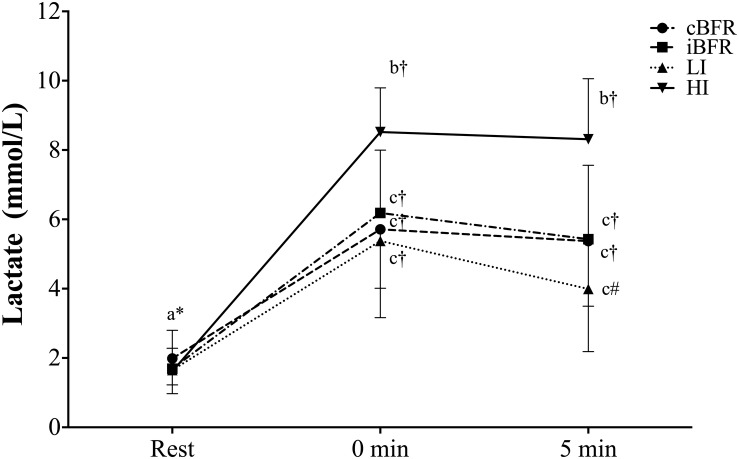
Absolute blood lactate concentration over time. cBFR, Low-load resistance exercise with continuous BFR; iBFR, low-load resistance exercise with intermittent BFR; LI, low-load resistance exercise without BFR; HI, high-load resistance exercise without BFR. Different letters denote significant condition differences, and different symbols denote significant time differences.

There was a significant condition-by-time interaction (*F* = 15.56, *p* < 0.001, η*_*p*_*^2^ = 0.55) for [La^–^] (Figure 3). Follow-up analyses revealed that no significant (*p* > 0.05) differences existed across the cBFR, iBFR, and LI protocols at rest, immediately post or 5 min post exercise, while HI was significantly (*p* < 0.001) greater than all exercise conditions at immediately post and 5 min post exercise, but not at rest (*p* > 0.05). In the comparisons across time within each condition, all trials significantly (*p* < 0.001) increased [La^–^] from rest levels immediately post and 5 min post exercise. There were no significant differences (*p* > 0.05) between immediately post- and 5-min post exercise measures for the cBFR, iBFR, and HI exercise conditions, while lower [La^–^] levels were observed 5 min post compared to immediately post exercise for the LI trial.

### Muscle Swelling

A significant condition-by-time interaction (*F* = 3.31, *p* = 0.003, η*_*p*_*^2^ = 0.65) was observed for muscle thickness. As outlined in Table 3, there were no significant (*p* > 0.05) differences between any of the four experimental conditions immediately post exercise. However, 5 min post exercise, LI was (*p* < 0.05) significantly lower than HI and cBFR; and at 15 min post exercise, LI and cBFR were significantly (*p* < 0.05) lower than HI. However, both 5- and 15-min post exercise differences were lower than the MD needed to be considered a real change. In the comparison across time within each condition, muscle thickness was significantly (*p* < 0.05) above rest levels from immediately post- to 15 min post exercise for cBFR, iBFR, and HI conditions; however, these differences were lower than the MD and, hence, not considered real changes. Regarding LI, muscle thickness was significantly (*p* < 0.05) elevated compared to rest levels immediately post and 5 min post exercise but returned to rest levels 15 min post exercise (*p* > 0.05); additionally, immediately post exercise measures were significantly (*p* < 0.05) greater than 15-min post exercise values.

**TABLE 3:** Absolute muscle thickness (cm) and thigh circumference (cm) values over time for each exercise condition.

	**Rest**	**0 min**	**5 min**	**15 min**
**Muscle thickness**				
cBFR	4.91 ± 0.67	5.44 ± 0.72*	5.40 ± 0.71*	5.27 ± 0.71^b^*
iBFR	4.98 ± 0.73	5.48 ± 0.77*	5.38 ± 0.76*	5.35 ± 0.76*
LI	5.02 ± 0.72	5.35 ± 0.80*	5.24 ± 0.76*	5.14 ± 0.78^†^
HI	5.03 ± 0.79	5.51 ± 0.74*	5.53 ± 0.75*	5.50 ± 0.69*
**Thigh circumference**				
cBFR	53.94 ± 5.10	54.25 ± 5.29	54.12 ± 5.15	53.90 ± 5.14
iBFR	53.77 ± 5.29	54.42 ± 5.42	54.36 ± 5.35	54.16 ± 5.46
LI	54.00 ± 0.05	54.42 ± 5.12	54.41 ± 5.34	54.30 ± 5.35
HI	54.02 ± 5.44	54.88 ± 5.25	54.91 ± 5.21	54.73 ± 5.35

cBFR: low-load resistance exercise condition with continuous blood flow restriction (BFR), iBFR: low-load resistance exercise condition with intermittent BFR, LI: low-load resistance exercise condition without BFR, HI: high-load resistance exercise condition without blood flow restriction. ^*b*^Significantly lower than HI. * Significantly different from Rest. ^†^Significantly different from 0 min.

Regarding thigh circumference, there was no significant condition-by-time interaction (*F* = 2.75, *p* = 0.32, η*_*p*_*^2^ = 0.09) or significant condition main effect (*F* = 10.50, *p* = 0.24, η*_*p*_*^2^ = 0.11), but there was a significant time main effect (*F* = 10.93, *p* < 0.001, η*_*p*_*^2^ = 0.58). Further analysis of the time main effect revealed that thigh circumference significantly (*p* < 0.05) increased from rest levels immediately post and 5 min post exercise and returned to rest levels (*p* > 0.05) within 15 min post exercise. Additionally, immediately post- and 5-min post exercise measures were significantly (*p* < 0.05) greater than 15-min post exercise values. However, none of these increases were greater than the MD.

### Hematocrit and Plasma Volume Changes

As displayed in Table 4, there was a significant condition-by-time interaction (*F* = 3.21, *p* = 0.002, η*_*p*_*^2^ = 0.20) for Hct. Pairwise comparisons revealed that the HI exercise condition resulted in significantly (*p* < 0.05) higher hematocrit values than the iBFR and LI conditions, but not cBFR immediately post exercise. At 5 min post exercise, HI was significantly greater than LI, while, at 15 min, no significant (*p* > 0.05) differences were observed across conditions. Regarding the changes in hematocrit values across time points within each exercise condition, immediately post exercise and 5-min post exercise measures were significantly (*p* < 0.05) greater than rest values for all conditions, except for HI that elicited significantly (*p* < 0.05) greater hemoconcentration immediately post exercise compared to 5-min post exercise values. Hct immediately post exercise were also significantly (*p* < 0.05) greater than those 15 min post exercise for all conditions. Finally, hemoconcentration at 15 min post exercise was significantly (*p* < 0.05) greater than 5-min post exercise measures for all conditions, except for LI.

**TABLE 4:** Absolute hematocrit (%) values and plasma volume changes (Δ%) over time for each exercise condition.

	**Rest**	**0 min**	**5 min**	**15 min**
**Hematocrit**				
cBFR	45.25 ± 1.00	48.50 ± 2.24*	47.79 ± 1.95*	45.68 ± 1.50^‡^
iBFR	44.50 ± 0.92	47.43 ± 1.74^a^*	46.14 ± 1.55*	44.96 ± 1.10
LI	45.25 ± 0.96	47.25 ± 1.33^a^*	46.61 ± 1.60^a^*	44.71 ± 1.14^‡^
HI	44.93 ± 1.00	49.68 ± 2.55*	48.00 ± 2.36*†	45.64 ± 1.77^‡^
**Plasma volume change**				
cBFR	–	−11.90 ± 7.59	−9.40 ± 6.83	4.21 ± 6.01^b†‡^
iBFR	–	−10.94 ± 5.35^b^	−6.22 ± 5.91^†^	−1.76 ± 3.90^a†‡^
LI	–	−7.55 ± 5.68^b^	−5.11 ± 5.92^b^	2.35 ± 5.28^†‡^
HI	–	−17.21 ± 6.83	−11.49 ± 6.44^†^	−2.83 ± 4.68^†‡^

cBFR: low-load resistance exercise condition with continuous blood flow restriction (BFR), iBFR: low-load resistance exercise condition with intermittent BFR, LI: low-load resistance exercise condition without BFR, HI: high-load resistance exercise condition without blood flow restriction. ^*a*^Significantly different from cBFR (*p* < 0.05). ^*b*^Significantly different from HI. * Significantly different from Rest. ^†^Significantly different from 0 min. ^‡^Significantly different from 5 min.

There was a significant condition-by-time interaction (*F* = 3.36, *p* = 0.005, η*_*p*_*^2^ = 0.21) for plasma volume changes. Follow-up analyses indicated that, while no significant (*p* > 0.05) differences existed between cBFR and iBFR, HI elicited significantly (*p* < 0.05) greater decreases in plasma volume immediately post exercise compared to iBFR and LI. At 5 min post exercise, HI induced significantly (*p* < 0.05) greater decreases in plasma volume compared to LI. Finally, at 15 min post exercise, significantly (*p* < 0.05) lower plasma volumes were observed for the iBFR and HI exercise conditions compared to cBFR. For time comparisons within conditions, similar responses were observed for the cBFR and iBFR protocols with the largest significant (*p* < 0.05) decreases in plasma volumes taking place immediately post and 5 min post exercise, which were also significantly (*p* < 0.05) lower than 15 min post exercise measures. For iBFR and HI conditions, the largest significant (*p* < 0.05) decreases in plasma volume were detected immediately post exercise, which were significantly lower than 5-min post exercise and 15-min post exercise assessments and with 5-min post exercise measures being significantly (*p* < 0.05) lower than 15-min post exercise measures.

## Discussion

The primary goal of this study was to compare the acute physiological responses of young males to a single session of lower-body resistance exercise with either cBFR or iBFR. The secondary goal was to compare the responses from both BFR exercise trials to those from traditional low- and high-load resistance exercises without BFR. Our findings refuted our hypothesis that greater physiological responses would be observed with cBFR exercise in comparison to iBFR, since no differences between both exercise conditions were observed for any of the physiological markers assessed. However, the results from this study partially supported our secondary hypothesis, as greater responses were observed with traditional high-load resistance exercise compared to the low-load exercise trials.

These results support findings of previous research that investigated muscle activity during cBFR and iBFR resistance exercises. Yasuda et al. (2013) had young males complete four sets (30–15–15–15 repetitions) of unilateral arm curls at 20% of 1-RM, with 160 mmHg of BFR, and observed no significant differences in muscle activity between the cBFR and iBFR resistance exercise protocols. However, the authors detected greater muscle activity during the two BFR exercise conditions compared to the low-load exercise trial without BFR during the last portions of the third and fourth sets of the exercise, whereas, in the current study, no significant differences were observed across any of the low-intensity exercise conditions at any time point. This discrepancy between the present results and those from Yasuda et al. (2013) may be due to exercise selection. The present study utilized bilateral lower-body exercises, while the aforementioned study only included a unilateral upper-body exercise. Moreover, the findings of the present study parallel previous work regarding the difference in muscle activity between both low-load BFR and the high-load resistance exercise trials, in that resistance exercise combined with BFR tends to induce lower muscle activity than conventional high-load resistance exercise (Jessee et al., 2019).

The largest muscle activity was observed with conventional high-load resistance exercise, which was also accompanied by the largest exercise-induced metabolic responses, expressed by changes in [La^–^]. Considering that both exercise load and changes in the intramuscular metabolic environment are known for influencing muscle activity during exercise (Suga et al., 2009, 2010), it is understandable that the HI exercise condition displayed the greatest muscle activity, since it also elicited the highest mechanical stress, exercise volume, and metabolic response. One important outcome of the current study that warrants further consideration is that similar changes in muscle activity and metabolic stress were observed across the cBFR and iBFR resistance exercise protocols. This result may suggest that the metabolic stress taking place during each set of the exercise is responsible for the changes in muscle activity, while the release of the pressure during the rest interval does not seem to affect this response. The lack of differences in muscle activity between cBFR and iBFR yields an important practical implication, as cuff deflation during the rest interval between sets may improve exercise tolerance and subsequent adherence, especially for clinical populations and older individuals, while still evoking similar responses from the positive exercise stimulus.

Exercise-induced muscle swelling has been suggested to potentially play a role as one of the mechanisms responsible for inducing the positive adaptations observed with BFR resistance training (Loenneke et al., 2012a) and, therefore, an important component assessed in the present study. Takarada et al. (2000b) reported that applying BFR in the absence of exercise twice a day (each BFR session consisted of five 5-min sets of an averaged ∼240 mmHg of BFR with 3 min of deflation between sets), over the course of 12 days, following anterior cruciate ligament reconstruction, attenuated muscle atrophy in comparison to the control group. Although no measures of muscle swelling were performed in the aforementioned study, later work from Loenneke et al. (2012b) demonstrated that the application of BFR in the absence of exercise using a similar protocol to that from Takarada et al. (2000b) induces significant acute increases in muscle thickness and decreases in plasma volume, while no metabolic stress or changes in muscle activity were observed. These findings indicate that muscle swelling may play a role in the intramuscular anabolic response. In the present study, the combination of resistance exercise with BFR elicited significant increases in muscle thickness, which were similar to those observed with traditional resistance exercise and which were also accompanied by paralleling shifts in plasma volume, indicating that muscle swelling possibly happened in the active muscle. The results from the present study also demonstrated that both cBFR and iBFR result in acute muscle swelling following exercise in a similar fashion compared to conventional high-load resistance exercise. Hence, our results also suggest that releasing the pressure of the cuffs during the rest periods between sets does not attenuate the exercise-induced muscle swelling response. These outcomes corroborate previous findings from our research group that also demonstrated similar levels of muscle swelling with both low-load resistance exercise with BFR and conventional high-load resistance exercise (Freitas et al., 2017). Yasuda et al. (2015) also observed similar changes in muscle thickness following four sets of low-load (20% of 1-RM) resistance exercise with and without BFR performed to failure. However, caution is needed when discussing the potential contribution of exercise-induced muscle swelling to long-term exercise adaptations. In a recent study, Nyakayiru et al. (2019) demonstrated that BFR only increases myofibrillar protein synthesis when combined with resistance exercise in recreationally young males and speculated that BFR, in the absence of exercise, may only induce myofibrillar protein synthesis in individuals experiencing disuse states, such as those studied by Takarada et al. (2000b).

The present study is not without limitations. The total restrictive pressure was measured with participants lying down, while each exercise was performed with participants in the seated position. This approach may have affected the precision of our method to measure the BFR pressure to be used during each protocol. In this regard, Sieljacks et al. (2018) reported that 40% of seated BFR pressure corresponds to about 50% of BFR measure with the participant lying down, specifically in the lower body. Nonetheless, even if the restrictive pressure utilized in the current investigation corresponded to approximately 40% of BFR, previous studies have shown similar chronic neuromuscular adaptations with 40 and 90% of occlusion during resistance training combined with BFR (Counts et al., 2016). However, it is not completely clear if the pressure utilized in this study was high enough to elicit adequate acute physiological responses. Furthermore, this study included untrained male participants, which makes it challenging to generalize these results to other populations, such as females, athletes, or clinical populations. Lastly, muscle swelling was not assessed directly but only estimated through measures of muscle thickness and changes in plasma volume.

## Conclusion

In conclusion, our findings indicate that deflating the restrictive cuffs during the rest periods between each set does not attenuate the physiological responses commonly observed with continuous BFR resistance exercise, including increased muscle activity, metabolic stress, and muscle swelling. Therefore, restricting the blood flow during the muscular contractions seems to be more important for the acute exercise-induced physiological responses than maintaining BFR during the rest intervals. Additionally, greater physiological responses were observed with traditional high-load resistance exercise without BFR, indicating that greater mechanical stress and exercise volume seem to induce greater physiologic stimulus compared to BFR exercise. Finally, the outcomes of the present study yield relevant practical applications. Our results indicate that the restrictive pressure of the BFR cuff may be decreased or completely released during the rest intervals between sets if necessary to diminish discomfort and facilitate exercise tolerability without compromising the short-term exercise-induced physiological responses.

## Data Availability Statement

The datasets generated for this study are available on request to the corresponding author.

## Ethics Statement

The studies involving human participants were reviewed and approved by the University of Oklahoma Institutional Review Board. The patients/participants provided their written informed consent to participate in this study.

## Author Contributions

All authors contributed to designing the study, analyzing and interpreting the data, and writing and proofreading the manuscript. All authors also approved the content of the manuscript’s final version.

## Conflict of Interest

The authors declare that the research was conducted in the absence of any commercial or financial relationships that could be construed as a potential conflict of interest.
